# The use of mobile health interventions for gestational diabetes mellitus: a descriptive literature review

**DOI:** 10.25122/jml-2020-0163

**Published:** 2021

**Authors:** Maryam Zahmatkeshan, Somayyeh Zakerabasali, Mojtaba Farjam, Yousef Gholampour, Maryam Seraji, Azita Yazdani

**Affiliations:** 1. Noncommunicable Diseases Research Center, School of Medicine, Fasa University of Medical Sciences, Fasa, Iran; 2. Department of Health Information Management, Health Human Resources Research Center, School of Management and Information Sciences, Shiraz University of Medical Sciences, Shiraz, Iran; 3. Clinical Research Development Unit, Valie-Asr Hospital, Fasa University of Medical Sciences, Fasa, Iran; 4. Medical School, Fasa University of Medical Sciences, Fasa, Iran; 5. Health Promotion Research Center, Zahedan University of Medical Sciences, Zahedan, Iran

**Keywords:** m-health, gestational diabetes mellitus, gestational diabetes mellitus

## Abstract

This study attempted to review the evidence for or against the effectiveness of mobile health (m-health) interventions on health outcomes improvement and/or gestational diabetes mellitus (GDM) management. PubMed, Web of Science, Scopus, and Embase databases were searched from 2000 to 10 July 2018 to find studies investigating the effect of m-health on GDM management. After removing duplications, a total of 27 articles met our defined inclusion criteria. m-health interventions were implemented by smartphone, without referring to its type, in 26% (7/27) of selected studies, short message service (SMS) in 14.9% (4/27), mobile-based applications in 33.3% (9/27), telemedicine-based on smartphones in 18.5% (5/27), and SMS reminder system in 7.1% (2/27). Most of the included studies (n=23) supported the effectiveness of m-health interventions on GDM management and 14.3% (n=4) reported no association between m-health interventions and pregnancy outcomes. Based on our findings, m-health interventions could enhance GDM patients' pregnancy outcomes. A majority of the included studies suggested positive outcomes. M-health can be one of the most prominent technologies for the management of GDM.

## Introduction

High-risk complications are estimated to occur in 10 percent of pregnancies, and the evidence reveals the growing rate of high-risk pregnancies [1]. One of the most common complications that can occur during pregnancy is gestational diabetes mellitus (GDM) [2, 3], accounting for more than 80% of diabetes cases during pregnancy [2]. GDM is found in 2% to 16% of all pregnancies [4], affecting about 150,000 pregnancies annually [5, 6]. GDM affects both mother and child [7] and poses the mother and child at risk of preeclampsia, cesarean delivery, congenital anomalies, fetal macrosomia, and the later development of type 2 diabetes [8]. Given the increasing prevalence of GDM, new challenges are developed for health care professionals in antenatal care [9]. High-risk pregnancies are often managed by hospitalizing the patients for days and sometimes even weeks, leading to an increased financial burden [10] and stress for the patients [11]. By proper monitoring, the risk and the disease costs will be reduced for a pregnant woman [12–14].

The recent advances in medical devices coupled with the development of intelligent sensors, Internet of Things (IoT), efficient telecommunication and information based smart decision support system (DSS), and m-health technologies have unlocked the door of ample opportunities for patients' remote monitoring and health parameters tracking, thus enabling a paradigm shift in maternal health care [13, 15].

Recently, the dramatic advancements of information and communication technologies (ICTs) in health care have led to the development of m-health, creating substantial improvement in the provision of health services [16]. M-health interventions have been developed along with technological advances [17]. The widespread adoption of mobile phone technologies offers a promising opportunity to promote diabetes care and self-management [18–20] by creating an active interaction between patients and healthcare professionals [9, 20].

The increasing storage capacity of mobile phones along with Wi-Fi accessibility represent the opportunity to offer mobile applications with the capabilities of tracking one or more health parameters such as glucose, diet, exercise and medication [19, 21–25]. Mobile phone text messaging has enabled the provision of timely access to health advice, prompt self-monitoring, and individual’s education about preventive health care services [26]. Immediate delivery of short messages or direct calls to individuals is facilitated by mobile phone. Patients can be reminded over the cell phone at the time of the blood glucose measurement or another event (medication), leading to improvement of glycated hemoglobin (HbA1c) levels and self-care regarding diet, medication, and exercise [20]. Following the emergent of m-health interventions in GDM, the development and evaluation of individual interventions have attracted more attention by the researchers. The previous systematic reviews either have focused on the effectiveness of m-health interventions [4, 16, 27–34] or a single condition [35–38]. However, minimal evidence has been provided on healthcare utilization or cost analyses.

Therefore, the present study was attempted to systematically review the effectiveness of GDM-related m-health interventions from different perspectives. Recently, m-health has been introduced as a novel approach in GDM management. The delivery of face-to-face support has been examined in previous research; however, little is known about the implementation of m-health as a possible alternative form of health service delivery for GDM patients. The aim of this descriptive literature review was to assess the evidence provided for or against the efficacy of m-health in GDM monitoring.

## Material and Methods

### Search strategy

We performed this study according to the Preferred Reporting Items for Systematic Reviews and Meta-Analysis (PRISMA) [39]. PubMed, Web of Science, Embase, and Scopus databases were searched from 2000 to July 10, 2018. The searches were not limited by language.

The searches were done using the keywords of mobile health (m-health) and GDM. For the purpose of this review, m-health was defined as the use of technology to provide healthcare for patients with GDM using the short message service (SMS), mobile applications, and telemedicine systems based on smartphones.

The stages for building the search query for the PubMed database are shown in Table 1. With respect to the instructions provided for each database, equivalent searches were then performed. Findings of each study were analyzed, relevant information was extracted, and the obtained data were synthesized. The objectives and findings of each study are summarized in Table 2.

**Table 1.** PubMed search query.

Step in search strategy	Search term
**1**	mobile health OR mobile OR mhealth OR m-health OR mobile technology OR smartphone OR mobile phone OR cell phone OR app OR applications OR short message service OR short message OR text message OR SMS
**2**	(Diabetes, Pregnancy-Induced) OR (Diabetes, Pregnancy Induced) OR (Pregnancy-Induced Diabetes) OR (Gestational Diabetes) OR (Diabetes Mellitus, Gestational) OR (Gestational Diabetes Mellitus)
**3**	2000/01/01:2018/11/15[dp]
**4**	1 AND 2 AND 3

**Table 2.** Reviewed papers’ characteristics and m-health interventions and results from studies.

Author, Year	Study objective	Study design	Type of mHealth medium				Outcome (main findings)
			Smartphone	Telemedicine system based on smartphones	SMS	App	
**Ping Yang, 2018 [44]**	The effect of WeChat platform-based treatment on the risk of perinatal complications among women with GDM.	Non-Randomized Study		*			WeChat platform-based treatment could effectively reduce FBG and 2h PBG levels and improve pregnancy outcomes.
**Skar, 2018 [45]**	The effect of smartphone app (the Pregnant+ app) on controlling blood glucose levels and receiving health and nutrition information in women with GDM.	RCT				*	Smartphone app could assist women with GDM to control their blood glucose and increased their confidence regarding self-management.
**Miremberg, 2018 [46]**	The impact of a smartphone-based daily feedback and communication platform on GDM patients' compliance, glycemic control, pregnancy outcome, and satisfaction	RCT	*				Smartphone-based technology could enhance the adherence to self-performed BG monitoring and glycemic control parameters such as mean blood glucose, off-target measurements, and the need for insulin treatment.
**Mackillo, 2018 [47]**	The use of a mobile phone-based real-time blood glucose management system to control GDM patients' blood glucose.	RCT	*				Remote monitoring of blood glucose is safe in women with GDM.
**Rigla, 2018 [48]**	The efficacy of smart mobile telemedicine in monitoring blood glucose of GDM patients.	Pilot Study		*			This decision support system was a feasible and well-accepted system for monitoring GDM.
**Kennelly, 2018 [42]**	The impact of a healthy lifestyle package using smartphone application technology on the prevalence of GDM in overweight and obese women.	RCT				*	This intervention could not decrease the prevalence of GDM.
**Johnson, 2018 [49]**	The effect of short messaging reminders on diabetes self-management in women with GDM	RCT			*		The use of daily text messages was acceptable for patients with GDM. In addition, an overall satisfaction with the messages and willingness to use the messages in future pregnancies and to recommend the messages to friends with GDM were obtained.
**Garnweidner-Holme, 2018 [9]**	The usefulness of culture-sensitive pregnant application for pregnant women with GDM according to health care professionals' perspectives	Qualitative study				*	M-Health intervention was a useful tool to improve the care provided by health care professionals to women with GDM.
**Peleg, 2017 [50]**	The MobiGuide’s feasibility and potential impacts on patients and care providers using two various clinical domains.	Pilot Study				*	This system has provided multiple benefits for both patients and physicians and increase the patients' sense of safety and involvement.
**Peleg, 2017 [51]**	The system’s feasibility and potential impacts on patients and care providers using two various clinical domains.	Pilot Study				*	MobiGuide’s is feasible for patients and clinicians and has led to high compliance to self-measurement recommendations and enhance the satisfaction of patients and care providers.
**McLean, A. 2017 [41]**	The efficacy of real-time smartphone data in improving clinical management and outcomes of women at GDM risk	Pilot Study	*				Real-time individual health and sensor data can be readily collected and analyzed efficiently while confidentiality is maintained; however, improved prediction of GDM was not obtained.
**Nicholson, 2016 [52]**	The efficacy of a web-based pregnancy and postpartum behavioral intervention in contributing women with GDM to control weight and glucose during pregnancy and the postpartum period.	RCT			*		The web-based behavioral intervention coupled with text messages and emails and tailored to the needs of women with GDM is feasible and well received by participants. This study also shows that GooDMoms can change the current paradigm of pregnancy care for women with GDM.
**Marko, 2016 [53]**	The feasibility of remote monitoring of patients for prenatal care using a mobile phone application and connected digital devices.	Prospective Observational Study				*	This intervention is feasible for prenatal care.
**Wickramasinghe, 2015 [54]**	The usefulness of mobile technology for supporting and enabling superior diabetes monitoring and management.	Case Study	*				Mobile technology is an appropriate choice to minimize costs and provide high-quality care.
**Jo, 2015 [55]**	The efficacy of an application providing tailored recommendations based on user's lifestyle and clinical data.	Development and Test Study				*	The GDM management knowledge and tailored recommendations provided in this study were beneficial for managing GDM.
**Van Ryswyk, 2015 [40]**	The effect of SMS reminder system on postpartum oral glucose tolerance test, fasting plasma glucose, and HbA1c completion.	RCT			*		The SMS reminder system cannot enhance postpartum OGTT, fasting plasma glucose, or HbA1c completion.
**Mohd Suan, 2015 [56]**	The prevalence and characteristics of patients who did and did not return for the OGTT and the reasons provided by women for failure to return for the OGTT test.	Cross-sectional Study			*		The prevalence of women who returned for the postpartum diabetic screening test was high. This study also provides valuable insights into several obstacles that render the return for the glucose tolerance test.
**Hirst, 2015 [57]**	Women’s satisfaction using the GDM-health system and their attitudes toward their diabetes care.	Pilot Study				*	GDM-health was acceptable and convenient for a large proportion of women.
**Teoh, 2014 [58]**	The efficacy of smartphones on GDM monitoring among Australian women.	RCT	*				The use of smartphones to support GDM self-management facilitates superior monitoring and management of GDM and supports the accountable care paradigm.
**Kaplan, 2014 [59]**	Efficacy of a mobile application and web-based system in promoting self-management of women with GDM.	Pilot Study				*	This mobile application and web-based system can promote self-management of women with GDM
**Grabosch, 2014 [60]**	The feasibility of a text message reminder system for pregnant women with diabetes from a low-income population, and its impact on adherence to a diabetes care regimen and subsequent glycemic control	RCT			*		The text message reminder system is feasible for pregnant women with diabetes from a low-income population and Text4baby can be used as an educational tool to improve outcomes in women with diabetes.
**Shivanath, 2014 [61]**	The feasibility of “Simple Telehealth” for women with gestational diabetes, patients’ treatment satisfaction with this intervention, and the economic benefit of this system in conjunction with routine antenatal diabetes care.	Pilot Study			*		Short-term use of “Simple Telehealth” is associated with high treatment satisfaction levels amongst patients with GDM and some economic benefits.
**Homko, 2012 [43]**	The impact of an enhanced telemedicine system on glucose control and pregnancy outcomes in women with GDM.	RCT		*			Enhanced telemedicine monitoring system increased contact between women with GDM and their healthcare providers but did not influence on pregnancy outcomes.
**Shea, 2011 [62]**	The effect of a reminder system on screening rates.	RCT	*				Reminders can be an effective method for reinforcing guidelines for postpartum diabetes screening.
**P′erez-Ferre, 2010 [63]**	The feasibility of a telemedicine system based on Internet and a short message service for pregnant women with GDM and its influence on their delivery and neonatal outcomes.	RCT			*		A telemedicine system can be a useful tool in the treatment of GDM patients. This study suggests this intervention as a complement to conventional outpatient clinic visits, especially in cases requiring tighter glycemic control or with difficulties in accessing to medical center.
**Wickramasinghe, 2010 [64]**	The effect of a wireless technology on management of GDM	Pilot Study	*				DiaMonD is a convenient, cost-effective, and superior intervention to manage GDM.
**Dalfra, 2009 [65]**	The effect of a telemedicine approach on diabetic pregnancy management, glycemic control, quality of life, and maternal and fetal outcomes.	Non-Randomized Study		*			The use of a telemedicine system for glucose monitoring has improved pregnancy outcome and quality of life in women with GDM

BG – blood glucose; FBG – fasting blood glucose; GDM – gestational diabetes mellitus; PBG – plasma blood glucose; RCT – randomized controlled trial; OGTT – oral glucose tolerance test.

### Inclusion and exclusion criteria

In this study, all articles evaluated the effectiveness of m–health on GDM management by using m-health tools such as telemedicine systems based on smartphones, m-Health, short message service (SMS), and mobile applications were included. Reviews and unpublished dissertations, commentaries, opinion papers, editorials, summaries were not considered. Studies that used telemedicine intervention without a mobile phone or those that reported functions and implementation of the interventions unclearly or presented descriptions of information technology were excluded.

### Selection of studies

The selection of studies was done using four stages and based on the PRISMA flow diagram [39]. In the first stage (identification), studies identified through database searching were collated using the ENDNOTE software, and duplicates were omitted. In the second stage, two reviewers independently screened the titles and abstracts and removed irrelevant articles.

In the third stage, the full-text articles were independently evaluated for eligibility by the reviewers. In the fourth phase, two reviewers (MZ and ZK) compared and verified their findings. Any disagreement was resolved either through discussion or involving a third reviewer (YGH). Data such as author names, year, study design, m-health intervention, outcome, and results were retrieved.

## Results

**Figure 1.**
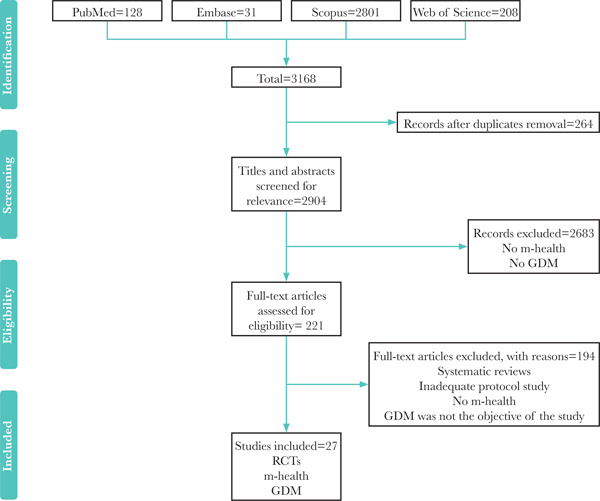
PRISMA flow diagram of search strategy. RCTs – randomized controlled trials; GDM – gestational diabetes mellitus.

The article selection process is demonstrated in Figure 1. Observing our defined inclusion and exclusion criteria, twenty-seven studies were selected: twelve randomized controlled trials (RCTs); eight pilot studies; one cross-sectional study; two non-randomized controlled trials; one case study; one prospective observational study; one qualitative study; and one development and test study. The findings of the included studies concerning the efficacy of m-health interventions are summarized in Table 2.

Among the 27 studies, 26% (7/27) used a smartphone, without referring to its type, 14.9% (4/27) used the SMS, 33.3% (9/27) used mobile-based applications, 18.5% (5/27) used telemedicine based on smartphones, and 7.1% (2/27) used an SMS reminder system to investigate the efficacy of m-health interventions. Often, these studies supported the efficacy of m-health interventions (n=4) [40–43].

## Discussion

To manage the disease and reduce the impact of chronic diseases, mobile health technologies can be helpful through the promotion of healthy behaviors [15, 66, 67]. These newly introduced technologies can be served as self-monitoring tools for individual patients [68–70] and can effectively enhance women’s health [71].

Literature reviews have supported the efficacy of mobile-health interventions in the management of diabetes, proving to be beneficial for patients, especially GDM patients (to control their blood sugar levels [20, 31, 72–80].

In the current investigation, all findings in this regard are presented using a structure composed of four main categories of m-health intervention. These four branches are:

1. *Smartphone-based:* Smartphones support various aspects of care and patient-clinician interactions, provide high-quality care and support self-management of GDM [46, 47, 54, 57, 58, 62]. The use of m-health can increase GDM patients’ compliance with lifestyle interventions and reduce future risk of type 2 diabetes mellitus and its sequelae [81]; however, this result was not supported by McLean *et al.* [41].
2. *Smartphone-based telemedicine system:* The impact of telemedicine interventions on GDM management has been investigated by many studies [43, 44, 48, 63, 65]. For instance, some studies have revealed the efficacy of telemedicine systems in monitoring glucose, improving pregnancy outcomes in women with GDM, and enhancing the quality of life of pregnant women with diabetes [44, 48, 63, 65]. These findings are in line with the results of other studies [4, 82, 83]. Nevertheless, Homko *et al.* [43] and Rasekaba *et al.* [82] found no association between a telemedicine system and pregnancy outcome improvement.
3. *SMS and reminders:* Studies have demonstrated the potential and essential role of SMS in altering the current paradigm of pregnancy care among women suffering from GDM [49, 52, 61]. Poorman *et al.* has supported the usefulness of SMS for maternal and infant health, especially for women who cannot outreach traditional communication methods [33]. Another study has revealed that Text4baby, a free mobile health information service delivering health-related SMS to pregnant and postpartum women, can enhance physical activity participation [84]. Given that most of the women suffering from GDM do not present for postpartum glucose testing despite recommendations, SMS can play an important role in increasing the postpartum return rate of women with GDM for diabetic screening tests [56]. However, Van Ryswyk *et al.* have not confirmed this finding [40].
4. *Mobile application:* Nine out of 27 selected studies have evaluated the efficacy of mobile applications. Eight have provided evidence supporting the usefulness of mobile applications for managing GDM [9, 45, 50, 51, 53, 55, 59, 64]. In contrast, Kennelly *et al.* [42], in line with two other studies [85, 86], have reported a lack of association between GDM management and mobile applications.

Patient satisfaction is one of the key factors in using mobile devices. We found that six studies [46, 47, 49, 51, 57, 61] have addressed the effect of m-health interventions on patient satisfaction, revealing higher satisfaction levels in pregnant women who received prenatal support via mobile phone. This finding is in line with the findings of previous studies [87, 88]. Kim *et al.* have also demonstrated the association between a high level of user satisfaction and using diabetes notepad application and the positive effect of this application on diabetes self-management [75].

Six studies have evaluated disease costs and reported that m-health interventions are economically cost-effective and can reduce disease costs [49, 51, 54, 58, 61, 64].

Considering the widespread use of mobile phones, various m-health tools have been developed for disease management and monitoring. However, the most effective tool for the management of GDM has not been reported yet. Therefore, in this systematic review, we have summarized the findings of previous studies on the effect of m-health interventions on GMD management.

The major limitation of this study is that only four databases were searched, which could have led to the missing of high-quality articles on m-health intervention for GDM. Published studies on GDM-related m-health interventions are increasing; however, the results are not consistent. Therefore, further evaluations are needed to obtain consistent conclusions regarding the usefulness of m-health interventions for GDM management. Future research is recommended to evaluate m-health interventions using multiple functions or stages, especially those popular outside clinical practice.

## Conclusion

Future research is recommended to evaluate m-health interventions using multiple functions or stages, especially those popular outside clinical practice. We consider that m-health intervention is one of the most important technologies for GDM management. Acknowledging the risks of gestational diabetes and the growing incidence of this disease, the risk and the disease costs could be reduced for a pregnant woman by proper monitoring. The widespread adoption of mobile phone technologies offers a promising opportunity to promote diabetes care and self-management by promoting healthy behaviors.

## Acknowledgments

### Conflict of interest

The authors declare that there is no conflict of interest.
